# Retraction: Prediction and optimization of employee turnover intentions in enterprises based on unbalanced data

**DOI:** 10.1371/journal.pone.0307474

**Published:** 2024-07-16

**Authors:** The PLOS ONE Editors

After this article [1] was published, the second author contacted the journal stating no knowledge of the first author nor of [1].

The corresponding author’s listed affiliation, Boston University Metropolitan College, stated that the study reported in [1] was not part of academic work carried out at this institution.

The *PLOS ONE* Editors further note that the datasets and code used in [1] have not been made publicly available as required by the PLOS Data Availability policy.

The corresponding author did not respond to correspondence about these issues.

In light of the above concerns about the integrity of the reported authorship and contribution information, the *PLOS ONE* Editors retract this article.

EF agreed with retraction. ZL either did not respond directly or could not be reached.
